# The Development of a Novel Sodium Alginate-Based Edible Active Hydrogel Coating and Its Application on Traditional Greek Spreadable Cheese

**DOI:** 10.3390/gels9100807

**Published:** 2023-10-07

**Authors:** Aris E. Giannakas, Konstantinos Zaharioudakis, Eleni Kollia, Anna Kopsacheili, Learda Avdylaj, Stavros Georgopoulos, Areti Leontiou, Vassilios K. Karabagias, George Kehayias, Efthymia Ragkava, Charalampos Proestos, Constantinos E. Salmas

**Affiliations:** 1 Department of Food Science and Technology, University of Patras, 30100 Agrinio, Greece; zacharioudakis.k@upatras.gr (K.Z.); sgeorgop@upatras.gr (S.G.); aleontiu@upatras.gr (A.L.); vkarampagias@upatras.gr (V.K.K.); gkechagi@upatras.gr (G.K.); effierag@yahoo.com (E.R.); 2 Laboratory of Food Chemistry, Department of Chemistry, National and Kapodistrian University of Athens Zografou, 15771 Athens, Greece; elenikollia@chem.uoa.gr (E.K.); akopsacheili@chem.uoa.gr (A.K.); leardaavdy@chem.uoa.gr (L.A.); 3 Department of Material Science and Engineering, University of Ioannina, 45110 Ioannina, Greece

**Keywords:** sodium alginate, active coating, thymol, halloysite nanotubes, hydrogels, barrier properties, antioxidant activity, traditional Greek spreadable cheese, preservation

## Abstract

The necessity of reducing the greenhouse effect by decreasing the carbon dioxide fingerprint directed the food packaging technology to use biobased raw materials. Alginates, which are derived from brown algae species, are one of the most promising biobased biopolymers for the development of edible active coatings capable of protecting food from oxidation/bacterial spoilage. In this study, sodium alginate, which was plasticized with glycerol and mixed with a biobased thymol/natural halloysite nanohybrid, was used to develop novel edible active coatings. Nanocomposite coatings were also developed in this project by mixing pure halloysite with sodium alginate/glycerol matrix and were used as reference material for comparison reasons. Instrumental analysis indicated a higher compatibility of a thymol/halloysite nanohybrid with a sodium alginate/glycerol matrix compared to pure halloysite with a sodium alginate/glycerol matrix. Increased compatibility resulted in improved tensile properties, water/oxygen barrier properties, and total antioxidant activity. These edible active coatings were applied to traditional Greek spread cheese and showed a reduction in the mesophilic microbial population over one log10 unit (cfu/g) compared to uncoated cheese. Moreover, the reduction in the mesophilic microbial population increased with the increase in halloysite and thymol content, indicating such sodium alginate/glycerol/thymol/halloysite hydrogels as promising edible active coatings for dairy products.

## 1. Introduction

The global effort to develop edible active packaging films and coatings arose from the invasion of the modern era’s spirit of sustainability and circular economy in the food packaging sector of the food industry [1,2,3,4,5,6]. In addition to common food packaging properties, an active packaging film and/or coating interacts with the food to enhance its preservation and shelf life [7,8,9,10,11]. In the food industry, the global environmental goal of reducing food waste and the CO_2_ fingerprint could be further approached by using edible active packaging films and coatings instead of films based on common fusel polymers.

Starch, chitosan, gelatin, pectin, and alginates are the most used biodegradable biopolymers for the development of edible active films and/or coatings [12,13,14,15,16,17,18]. The alginate chemical group consists of alginic acid and alginate salts. Such chemicals are used as food additives with epsilon food coding. Sodium alginate is the (E401) food additive, while potassium alginate is the (E402), (E403) is the ammonium alginate, and calcium alginate is the (E404). These alginates naturally occur in digestible polysaccharides commonly produced and refined from the various genera of brown algae. Alginates are composed of the unbranched, linear, binary copolymers -D-mannuronic acid (M) and -L-guluronic acid (G), which are linked by 1–4 glycosidic bonds [16,19]. The water solubility of alginic acid and calcium alginate is practically zero, while the polymers sodium alginate, potassium alginate, and ammonium alginate are completely dissolved in water. Alginates are considered the most promising candidates for edible active food packaging films and coatings in the food packaging sector. The main reason for this is that alginic acid and its salts (E400–E404) are included in the lists of the European Commission (EC) for approved food additives. Thus, alginates are widely used in various food industries for many purposes, e.g., as thickening agents, stabilizers, emulsifiers, chelating agents, encapsulations, swelling agents, suspending agents, etc. Furthermore, they are used for gel, film, and membrane formation [19,20]. Among all alginates, sodium alginate is the most commonly used salt for making edible films and coatings [19].

Essential oils (EOs) and their derivatives are phytochemicals which, in the last decade, have been widely applied in active food packaging due to their natural abundance and significant antioxidant and antimicrobial activity [21,22,23]. The volatile nature of EOs enhances their evaporation and reduces their antioxidant/antimicrobial activity. To avoid this phenomenon, their adsorption on low-cost and naturally abundant nanoclays such as bentonite and halonite was proposed. EOs/nanoclay hybrid materials can control the release of EOs molecules when added to an active food packaging film [24,25]. Halloysite nanotubes (HNTs) belong to kaolin groups and are aluminosilicate, clay-based nanotubes. HNTs are some of the most used nanoclays for the production of active food packaging materials [25]. HNTs are typically 0.2–1.5 μm in length, while the inner and the outer diameters are ca. 10–30 nm and 40–70 nm, respectively. HNTs are nanostructured materials (at least one dimension in the range of 1–100 nm) [26]. Their advantages are the lack of toxicity, their low cost, and their biocompatibility. Their unique tubular structure makes an HNT a desirable nanofiller material for sustainable packaging and a unique carrier for active compounds such as EOs and other phytochemicals.

Tsalafouti is an artisanal traditional Greek cheese, with the possibility of being nominated as a Protected Designation of Origin (PDO) product and able to significantly stimulate local entrepreneurship [27]. The traditional Greek methods of cheese production are multiple and diverse. Tsalafouti stands out for its unique preparation method and for its sensory properties [28]. Produced locally in the Greek mountains, using ovine milk during the late summer lactation period, Tsalafouti is an acidic curd that has a smooth, spreadable texture; a solid white color; and a mildly acidic, sour, and refreshing taste [29]. The production process is based on spontaneous acid fermentation by indigenous microorganisms since no starter culture or rennet is added. Traditionally, this cheese was stored in caves under running water for several days to acidify. Its compact texture usually originates through the isoelectric precipitation of casein micelles due to the decrease in pH. This process begins with the milk’s native microorganisms under the microclimate of the maturation environment [22]. Recently, Pappa et al. [24] produced traditional “Tsalafouti” and studied physicochemical, microbiological and sensorial characteristics throughout the ageing of artisanal “Tsalafouti” [24]. According to their results from their microbiological analysis as well as mainly from sensory panel evaluations, “Tsalafouti” can have a shelf life of 45 days when stored aerobically in the refrigerator [24]. As far as we know, there is no study available considering the microbiological preservation of the commercial Greek spreadable cheese “Tsalafouti” after opening its package and keeping it under refrigerated conditions.

A recent study presented the development of a thymol-rich TO@HNT nanohybrid via a green distillation-evaporation/adsorption process [30]. The developed TO@HNT hybrid nanostructure has recently been successfully incorporated into a chitosan/polyvinyl alcohol matrix and low-density polyethylene (LDPE) matrix [30,31]. Such chitosan/polyvinyl-alcohol/ TO@HNT coatings and LDPE/TO@HNT active films were successfully tested in kiwi fruit preservation and fresh pork fillet preservation, respectively [30,31]. In this work, the performance of such TO@HNT hybrid nanostructures in a sodium alginate/glycerol (ALG/G) matrix was studied. A TO@HNT nanohybrid was homogeneously dispersed into an ALG/G aquatic solution at 5, 10, and 15 wt.% final nominal concentration to obtain ALG/G/xTO@HNT (x = 5, 10 and 15) active films/coatings. Pure HNT was also incorporated into an ALG/G aquatic solution at 5, 10 and 15 wt.% final nominal concentration to obtain active ALG/G/xHNT films/coatings (x = 5, 10 and 15) for comparison. Both the obtained ALG/G/xHNT and ALG/G/xTO@HNT films were characterized physiochemically with X-ray diffraction (XRD) analysis and Fourier Transform Infrared (FTIR) spectroscopy. Tensile and water/oxygen barrier properties as well as the antioxidant activity of the obtained films were also studied. Such tensile water/oxygen barrier and antioxidant activity properties are of great importance for the performance of such films as active coatings to prevent foods from oxidative and microbiological deterioration. Furthermore, the obtained ALG/G/xHNT and ALG/G/xTO@HNT films were applied for the first time as a new active coating to prevent the microbiological spoilage of commercial “Tsalafouti” cheese after opening its package. The specific points of the innovation of this study that are reported for the first time are as follows: (i) The preparation and characterization of such novel ALG/G/xTO@HNT active films (ii) and the application of such novel ALG/G/xTO@HNT films as edible active coatings on the commercial “Tsalafouti” cheese to extend its shelf life after the first opening of its package.

## 2. Results and Discussion

### 2.1. XRD Analysis of ALG/G/xHNT and ALG/G/xTO@HNT Films

**Figure 1:**
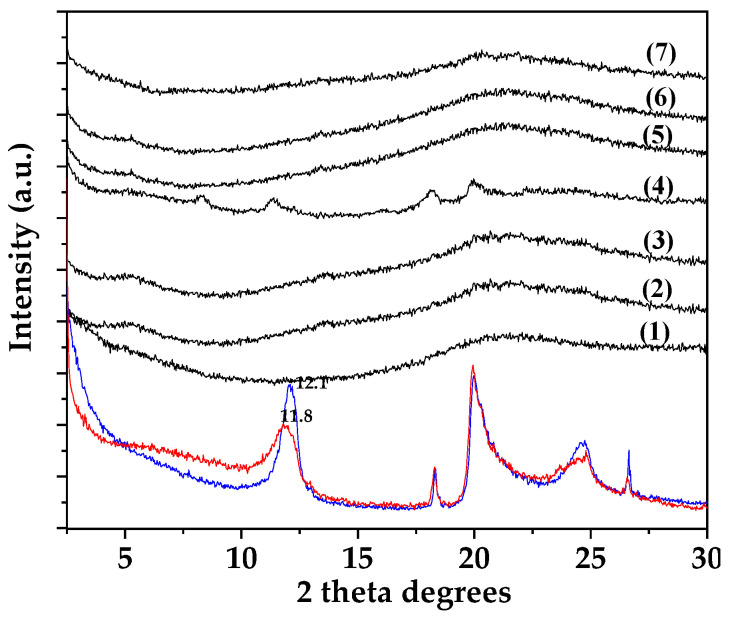
XRD plots of pure HNT (blue line) and modified TO@HNT nanohybrid (red line) as well as for pure ALG/G film (line (1)), ALG/G/5HNT film (line (2)), ALG/G/10HNT film (line (3)), ALG/G/15HNT film (line (4)), ALG/G/5TO@HNT film (lines (5)), ALG/G/10TO@HNT film (lines (6)), and ALG/G/15TO@HNT film (lines (7)).

Figure 1 shows the XRD plots of pure HNT powder (blue line) and TO@HNT nanohybrid powder (red line) in the 2–30° 2 theta range. In the same Figure 1, the XRD plots of pure ALG/G film (line (1)), all ALG/G/xHNT films (lines (2), (3), and (4)), and all ALG/G/xTO@HNT films (lines (5), (6), and (7)) are also shown for comparison.

According to previous reports [30,31], in both pure HNT and TO@HNT XRD plots (see blue and red line plots in the bottom of Figure 1), characteristic diffraction peaks at 2θ = 12.0, 20.1, and 24.6 2 theta correspond to the (001), (100), and (002) planes of a Halloysite nanotube crystal structure [30]. A slight decrease approx. 0.03° of HNT basal spacing probably indicates the insertion of small water molecules into the HNT’s interlayer space [30].

In the case of the pure ALG/G film XRD plot (see line (1) plot in Figure 1), the broad peak centered at 2θ = 21.6° corresponds to the amorphous structure of alginate [32].

In the case of all ALG/G/xHNT and ALG/G/xTO@HNT films XRD plots, no change in the characteristic peak of ALG/G at 21.6° is observed, implying that the addition of both HNT and modified TO@HNT do not affect the amorphous phase of the ALG/G matrix. When advanced, the characteristic peaks of the HNT crystal structure disappeared in all cases except in the case of the ALG/G/15HNT film. This indicates the high dispersity of both HNT and TO@HNT in the ALG/G matrix. A higher dispersity is achieved by the modified TO@HNT nanohybrid in comparison to the pure HNT [30].

### 2.2. FTIR Spectroscopy of ALG/G/xHNT and ALG/G/xTO@HNT Films

**Figure 2:**
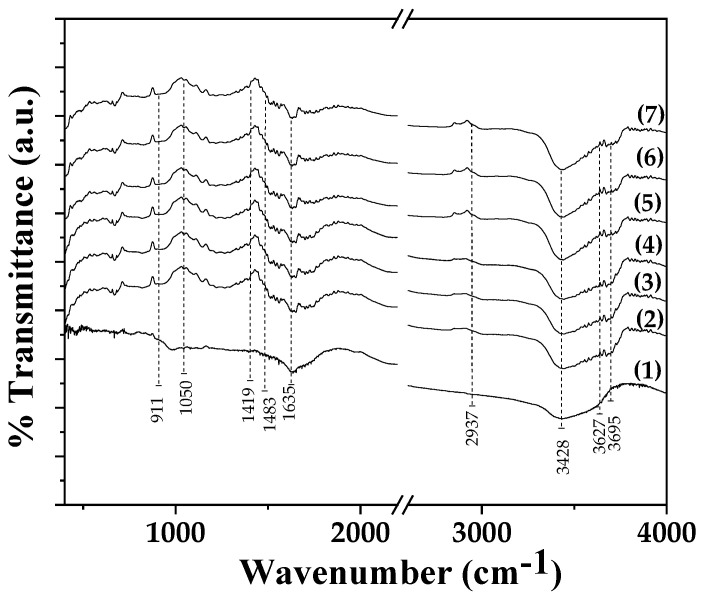
FTIR plots of (1) ALG/G, (2) ALG/G/5HNT, (3) ALG/G/10HNT, (4) ALG/G /15HNT, (5) ALG/G /5TO@HNT, (6) ALG/G/10TO@HNT, and (7) ALG/G /15TO@HNT obtained films.

FTIR plots of pure ALG/G film as well as from all ALG/G/xHNT and all ALG/G/xTO@HNT films are shown in Figure 2 for comparison.

In all plots, the characteristic peaks of sodium-alginate are observed. A broad band at 3428 cm^−1^ is assigned to the stretching vibration of hydrogen-bonded O–H groups [24,25]. A band at 1635 cm^−1^ is assigned to the asymmetric stretching vibration of COO groups, a band at 1419 cm^−1^ is assigned to the symmetric stretching vibration of COO groups, and a band at 1050 cm^−1^ is assigned to the elongation vibration of C-O groups [32,33].

In the case of HNT- and TO@HNT-based films in FTIR plots, the characteristic peaks of HNT are also observed among with sodium alginate bands. More specifically, a band at 911 cm^−1^ is assigned to the bending vibration of the Al–O–OH group bonds of HNT [34,35,36]. A band at 3695 cm^−1^ is assigned to the stretching vibration of the O-H group bond of HNT [35,36]. In addition, a peak at 1483 cm^−1^ is assigned to the deformation vibration of the Si-C bond of HNT, a band at 2937 cm^−1^ is assigned to the stretching vibration of C-H bond of HNT, and a band at 3627 cm^−1^—to the stretching vibration of the N-H bond of HNT [35,36].

With a careful glance, what is observed is that the main difference between the FTIR plots of ALG/G/xHNT and ALG/G/xTO@HNT is that in the case of all ALG/G/xTO@HNT FTIR plots, the band of the O-H group stretching at 3428 cm^−1^ is more intense than the same band of all the ALG/G/xHNT FTIR plots. This implies a higher interaction between OH groups of the ALG/G matrix and OH groups of the modified TO@HNT nanohybrid than OH groups of the ALG/G matrix and pure HNT OH groups. Thus, FTIR in accordance with XRD suggested a higher interplay/dispersion of the modified TO@HNT nanohybrid in the ALG/G matrix than pure HNT in the ALG/G matrix [30].

### 2.3. Tensile Properties of ALG/G/xHNT and ALG/G/xTO@HNT Films

The calculated Elastic Modulus (E), ultimate strength (σ_uts_), and %elongation at break (ε%) values of all ALG/G/xHNT and ALG/G/xTO@HNT films as well as pure ALG/G film are listed in Table 1 for comparison.

As can be seen from Table 1 and in the case of ALG/G/xHNT, the addition of 10 and 15 wt.% pure HNT content in the ALG/G matrix leads to a significant increase in stress and strength values and a decrease in elongation at break values. This behavior is indicative of the successful addition of nanoclay in polymer/biopolymer matrix, which results in nanocomposite films. In the case of adding 5 wt.% HNT to the ALG/G matrix, a significant reduction in stress and strength values is achieved in combination with a significant increase in %elongation at break values. Although these results are somewhat peculiar, similar trends have been previously documented on the addition of a sodium montmorillonite (NaMMT) nanoclay to a chitosan/glycerol matrix [37]. It has been established that the addition of 2.5 wt.% NaMMT was associated with the more homogenous distribution of water and glycerol across the system, resulting in a better plasticization effect in resulting nanocomposite films. On the contrary, in the case of all ALG/G/xTO@HNT films, the addition of all wt.% contents of TO@HNT results in nanocomposite films with higher stress-strength values than a pure ALG/G film. In addition, the obtained %elongation at the break value of the ALG/G/5TO@HNT film is higher than the ALG/G film, while for ALG/G/10TO@HNT and ALG/G/15TO@HNT films, the %elongation at break values are lower than the ALG/G film. Moreover, for ALG/G/10TO@HNT and ALG/G/15TO@HNT films, the %elongation at break values are higher than the %elongation at the break values of ALG/G/10HNT and ALG/G/15HNT films, respectively. Thus, combining the tensile property results shows that the addition of TO@HNT to the ALG/G matrix leads to “stronger” nanocomposite films consistent with the higher interaction of TO@HNT with the ALG/G matrix mentioned above in the FTIR and XRD characterization results. It is also obvious that the TO molecules act as plasticizer, which boosts the plasticization of obtained nanocomposite films [38].

### 2.4. Water/Oxygen Barrier Properties ALG/G/xHNT and ALG/G/xTO@HNT Films

In Table 2, the calculated *WVTR* and OTR values of all ALG/G/xHNT and all ALG/G/xTO@HNT films as well as pure ALG/G film are listed. From these values, the Water Vapor Diffusion coefficient (*D_wv_*) and oxygen permeability (Pe_O2_) values are calculated and listed for comparison.

As can be deduced from the *D_wv_* values in Table 2, the Water Vapor Diffusion coefficient of ALG/G/xHNT and ALG/G/xTO@HNT films is decreased compared to the *D_wv_* value of pure ALG/G film except for the *D_wv_* value of ALG/G/5HNT film. A higher *D_wv_* value decrease is achieved for TO@HNT-based films than HNT-based films due to the hydrophobic nature of TO. The lowest *D_wv_* values are achieved from ALG/G/10TO@HNT and ALG/G/15TO@HNT films.

The Pe_O2_ values of all ALG/G/xHNT films and all ALG/G/xTO@HNT films are lower than the Pe_O2_ values of the pure ALG/G film. ALG/G/xTO@HNT films achieve the lowest Pe_O2_ values than the ALG/G/xHNT films. The lowest Pe_O2_ values are achieved from ALG/G/10HNT and ALG/G/10TO@HNT films.

### 2.5. DPPH Assay Total Antioxidant Activity Values ALG/G/xHNT and ALG/G/xTO@HNT Films

**Figure 3:**
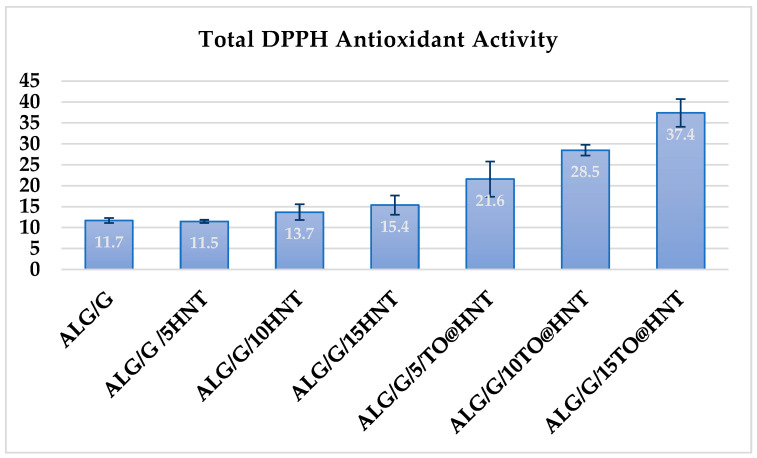
The total calculated DPPH antioxidant activity values of the films.

In Figure 3, the total antioxidant activity values calculated for all ALG/G/xHNT and all ALG/G/xTO@HNT films as well as the pure ALG/G film are listed for comparison.

Small antioxidant activity values are obtained for all ALG/G/xHNT films as well as for the pure ALG/G film due to the antioxidant properties of sodium alginate chain. All the TO@HNT-based films exhibited significant antioxidant activity due to the presence of TO molecules. Antioxidant activity of ALG/G/xTO@HNT film increased as the wt.% content of TO@HNT increased.

### 2.6. Application of ALG/G/xHNT and ALG/G/xTO@HNT as Active Coatings to Preserve “Tsalafouti” Type Spreadable Cheese

**Figure 4:**
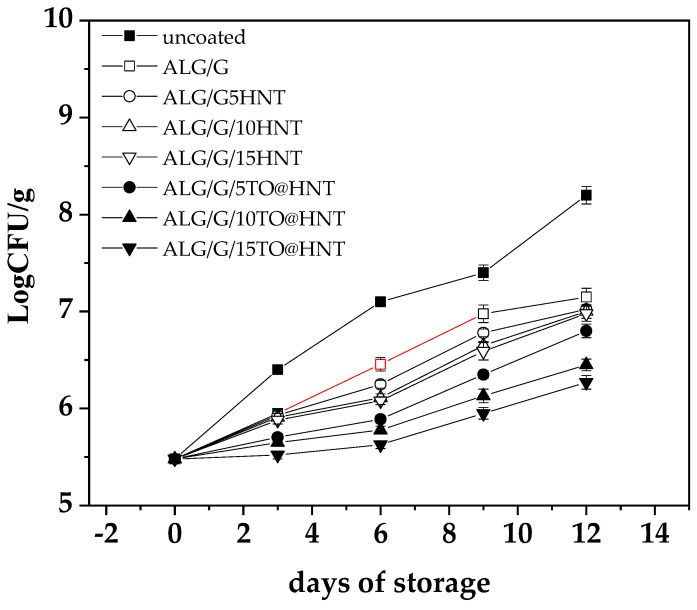
Comparison of Microbial Growth in Tsalafouti Cheese with Coatings Over 12 Days.

All ALG/G-based films tested above were used as active coatings to assess their efficacy in protecting “tsalafouti” spreadable cheese against mesophilic microbial population growth for 12 days after the opening of the commercial plastic packaging. Throughout the 12-day experimental period, microbial enumeration for different “Tsalafouti” cheese samples, both coated and uncoated, demonstrated a discernible trend. All samples recorded an escalation in log10 cfu/g values, a marker indicative of bacterial proliferation. The uncoated sample exhibited the most pronounced growth, escalating from an initial 5.48 log10 cfu/g to a final count of 8.20 log10 cfu/g on Day 12 (Figure 4).

As we can see in Figure 4 the uncoated “Tsalafouti” sample shows total microbial growth values greater than 7 log_10_ cfu/g after the sixth day of storage. This value (7 log_10_ cfu/g) is considered the upper TVC microbiological limit for the acceptable quality of foods according to the ICMSF [39]. This result agrees with the label on the commercial plastic packaging, which informs consumers that they must eat this product 3 to 4 days after opening it, thus validating the results of our microbiological analysis.

For the coated samples, the upward trend persisted, albeit with variable rates of microbial proliferation dependent on the specific coating type. The final log10 cfu/g values for ALG/G, ALG/G/5HNT coatings ranged between 7.00 and 7.02. Despite their continued growth, the ALG/G/10HNT and ALG/G/15HNT coatings yielded marginally lower final values, 7.00 and 6.98, respectively. The ALG/G/5TO@HNT and ALG/G/10TO@HNT coatings demonstrated a more controlled rate of microbial growth, with final log10 cfu/g values falling between 6.80 to 6.45 on day 12 of storage. Most notably, the ALG/G/15TO@HNT coating culminated with the lowest final microbial count, reporting a log_10_ cfu/g value of 6.27. This result posits the ALG/G/15TO@HNT coating as the most effective in managing bacterial proliferation among all tested coatings. Such an ALG/G/15TO@HNT coating could potentially be used as an novel alternative coating in the inner surface of commercial polypropylene-based packaging to protect this traditional kind of soft cheese and extend its preservation after opening from 3–4 days up to 12 days.

Notably, the results reflect a reduction in more than one log_10_ cfu/g unit across all samples when comparing the initial microbial count to the anticipated growth in the absence of coating. This finding indicates the significant role of the coatings in inhibiting microbial growth in Tsalafouti cheese over time. The results are in line with other relative studies which demonstrated that alginate sodium coatings enhanced with clays or essential oil offer a log10 cfu/g reduction [32]. Additionally, the trend of the growth of microorganisms in the coated “Tsalafouti” samples is comparable to the trends of the water/oxygen barrier properties of films. It is known that oxygen availability and water activity on the surface of cheese is crucial for the growth of microorganisms. Thus, the increased water/oxygen barrier properties of the obtained ALG/G/xHNT and ALG/G/xTO@HNT films decreased the growth of microorganisms.

Moreover, the results presented in Table 3 illustrate the pH changes in Tsalafouti soft cheese during 12 days of storage at 8 °C, under various alginate-based coatings enriched with halloysite nanotubes (HNT) and thyme oil (TO), applied at levels of 5, 10, and 15%. Across all samples, there is a noticeable decrease in pH, indicative of the production of galactic acid. The uncoated cheese displayed the most significant reduction in pH, whereas the alginate-based coatings resulted in a more gradual decline. Among the HNT and TO coatings, higher concentrations tended to maintain slightly higher pH levels. This pattern might imply a potential delay in microorganism population growth, especially at 10 and 15 wt.% contents of HNT and thyme oil. These findings emphasize the role of the coating type and concentration in influencing pH and by extension, microbial stability, offering a promising avenue for optimizing cheese preservation and quality.

The pH of the uncoated cheese, which served as the control in this experiment, decreased from 4.59 on day 0 to 4.315 on day 12. The decrease in pH indicates an increase in acidity, which is frequently associated with the proliferation of lactose-consuming microorganisms, such as lactic acid bacteria. As these microbes metabolize lactose, they produce lactic acid, lowering the pH and thereby indicating their proliferation and growth.

During the same 12-day period, all the coated cheese samples maintained higher pH levels, indicating less microbial activity. The specific coatings appear to effectively inhibit the growth of microorganisms, as evidenced by the smaller pH decrease observed in coated cheese compared to uncoated cheese. Among the coated samples, the ALG/G/15HNT, ALG/G/10TO@HNT, and ALG/G/15TO@HNT coatings maintained the highest pH levels on day 12, indicating that these coatings may be the most effective at inhibiting microbial growth, if all other factors remain constant. The findings of this research are consistent with prior work, specifically Silva et al. (2022), where similar effects were observed in alginate-coated and uncoated fresh cheese samples [40]. This reinforces the potential applicability of alginate-based coatings in extending cheese shelf life.

## 3. Conclusions

Apart from the circular economy and environmental benefits, which will be achieved by the development of such food packaging and coatings, a qualitative novel product exhibiting improved mechanical, water/oxygen barrier, antioxidant, and antimicrobial activity properties was developed during this study. The optimum sample, ALG/G/15TO@HNT, which was indicated as the most promising material, could be the target of a next step, scaled-up, and similar process. The results from the preservation of a very sensitive food such as the Greek soft cheese “Tsalafouti” showed that we have prepared a material potentially capable of being applied as an edible active packaging or coating in a wide variety of dairy products.

## 4. Materials and Methods

### 4.1. Materials

Acros-Organics (Zeel West Zone 2, Janssen Pharmaceuticalaan 3aB2440 Geel, Belgium) was the supplier of the sodium alginate powder, while Carlo-Erba (Denzlinger Str. 27, 79312 Emmendingen, Germany) was the provider of the glycerol. Chemco (Via Achille Grandi, 13–13/A, 42030 Vezzano sul Crostolo RE, Italy) was the producer of the thyme oil and a local pharmacy market was the supplier. The halloysite clay nanotubes used were purchased by Sigma-Aldrich (product 685445, Sigma-Aldrich, St. Louis, MO, USA). Fifteen fresh pork pancetta with an approximate weight of 400 g each were provided by a local meat processing plant—Aifantis Company—within one hour after slaughtering. The media used for microbiological analyses were plate count agar (PCA) and Mueller Hinton Agar (MHA). These media were purchased from VWR International GmbH. “Tsalafouti” traditional soft cheese was purchased from a local market, and it is a product of the “Papathanasiou” local dairy products factory. Τhis commercial “Tsalafouti” cheese was packaged in a polypropylene plastic package and was labeled “eat best before three to four days after opening”.

### 4.2. TO@HNT Hybrid Nanostructure Preparation

The rich-in-thymol TO@HNT hybrid nanostructure used was prepared according to the methodology recently described, which is based on a novel green distillation-evaporation/adsorption process [30,31]. According to the physicochemical characterization of this TO@HNT nanohybrid with an XRD analysis, FTIR spectroscopy, thermogravimetric (TG) analysis, and differential scanning calorimetry (DSC) measurements of pure HNT and a modified TO@HNT nanohybrid, it was shown that the physisorption of a mixture rich in TO molecules took place on the external surface of HNT, while the calculated average TO content on HNT was 34.5 wt.% [30,31]. In such EO–nanoclay hybrids, the controlled release process of the physiosorbed molecules was easier compared to the relevant processes of the chemisorbed molecules.

### 4.3. Preparation of ALG/G/xHNT and ALG/G/xTO@HNT Active Films

**Figure 5:**
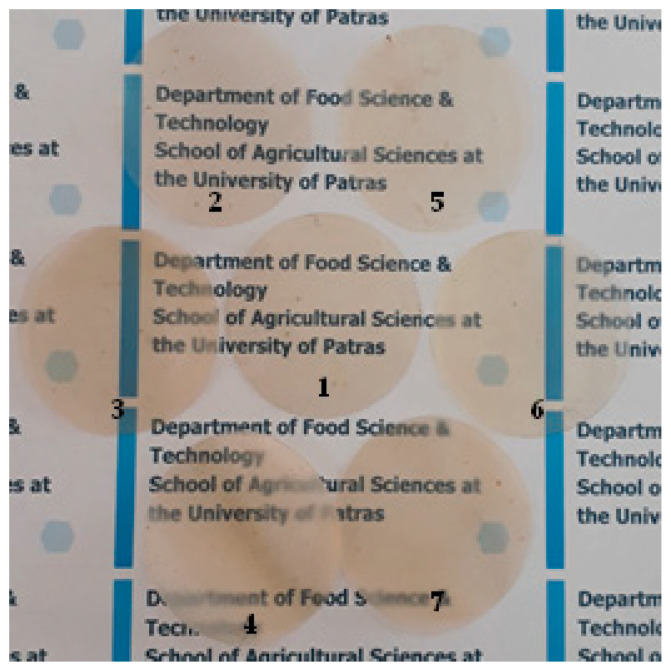
Photo images (1) pure CS/PVOH films, (2) CS/PVOH/5HNT, (3) CS/PVOH/10HNT, (4) CS/PVOH/15HNT, (5) CS/PVOH/5TO@HNT, (6) CS/PVOH/10TO@HNT, and (7) CS/PVOH/5TO@HNT films.

For each film, an amount of 2 g ALG was diluted in 90 mL distilled water with 1 g of G. The aqueous solution was heated at 100 °C under continuous stirring at 1000 rpm and for 1 h until a homogeneous hydrogel was obtained. Heating occurred at 100 °C and stirring occurred at 1000 rpm in an open glass beaker support aeration during the mixing process, avoiding the formation of foam. Simultaneously, 0.15, 0.30, and 0.45 g of pure HNT powder or TO@HNT nanohybrid powder was dispersed under vigorous stirring in 10 mL of distilled water using a glass beaker. Then, the homogeneous ALG/G hydrogel was gradually added to the obtained HNT or TO@HNT suspension and vigorously stirred at 2000 rpm for 2 h. Two Petri dishes (approx. 50 mL each), 11 cm in diameter, were used to spread the obtained ALG/G/xHNT (where x = 5, 10 and 15) and ALG/G/xTO@HNT (where x = 5, 10 and 15) hydrogels. The dishes were dried at 25 °C to obtain the final films. For comparison reasons, a pure ALG/G film without the addition of HNT powder or TO@HNT nanohybrid was prepared the same way. All final films were further stored at 25 °C and 50% RH in a desiccator (see Figure 5).

### 4.4. XRD Analysis of ALG/G/xHNT and ALG/G/xTO@HNT Films

A Brüker D8 Advance X-ray diffractometer instrument (XRD, Brüker, Analytical Instruments, S.A., Athens, Greece) was used to characterize the obtained ALG/G/xHNT, ALG/G/xTO@HNT, and pure ALG/G films. Analysis parameters and conditions were recently described [30].

### 4.5. FTIR Spectroscopy of ALG/G/xHNT and ALG/G/xTO@HNT Films

An FT/IR-6000 JASCO Fourier transform spectrometer (JASCO, Interlab, S.A., Athens, Greece) was used to investigate the relaxations of HNT and TO@HNT materials with the ALG/G matrix. FTIR spectroscopy measurements were carried out via setting up experimental parameters and conditions as recently described [30].

### 4.6. Tensile Measuraments of ALG/G/xHNT and ALG/G/xTO@HNT Films

A Simantzü AX g 5kNt instrument (Simandzu Asteriadis, S.A., Athens, Greece) was used to measure the tensile properties of ALG/G/xHNT, ALG/G/xTO@HNT, and pure ALG/G films. Analysis was conducted following the ASTM D638 method specifications and according to the methodology recently described [41].

### 4.7. Water Vapor Transmission Rate Measurements and Water Diffusion Coefficient Calculation

The Water Vapor Transmission Rate (*WVTR* g/cm^2^·s) for all obtained ALG/G/xHNT and ALG/G/xTO@HNT films, as well as the pure ALG/G film, was measured according to the ASTM E96/E 96M-05 method at 38 °C and 95% RH by using and employing a handmade apparatus. The calculated *WVTR* values were transformed into water vapor diffusivity (*D_wv_*) values according to the theory and equations described in detail in previous publications [32,42]. Briefly, films of each sample that were 2.5 cm in diameter were placed on the top of a one-open end cylindrical tube made of plexiglass, which contained dried silica gel inside and was sealed by a rubber O-ring. The test tube was placed in a thermostatic humidity chamber at 38 °C and 98% relative humidity (RH). The test tubes were weighed periodically for 24 h, and the *WVTR* [g∙cm^−2^∙s^−1^)] was calculated according to the following equation:

(1) $$WVTR=\frac{\Delta G}{t\cdot A}$$

where: Δ*G* (g) is the increase in the weight of the tested tubes, *t* (s) is the passing of time, Δ*G*/*t* (g/s) is the water transmission rate through the film, which is calculated by the slope of the linear function Δ*G* = *f*(*t*), and *A* (cm^2^) is the permeation area of the film. Additionally, the weight of the tested films was measured before and after the *WVTR* test to exclude any absorption phenomena of humidity by the film.

For the diffusion process through a membrane, Fick law [43] was used to calculate the specific mass flow rate via the following equation:

(2) $$\frac{J}{A}=D\frac{\Delta C}{\Delta x}$$

where *J* (g/s) is the mass flow rate of a component through the membrane, *A* (cm^2^) is the membrane cross-sectional area permeated by this component, Δ*C* (g/cm^3^) is the concentration gradient of this component in the two sides of the membrane, and Δ*x* (cm) is the membrane thickness.

Assuming that in our apparatus, the silica gel on one side totally absorbs the permeated water vapor and given that, according to the ASTM E96/E 96M-05 method, the humidity concentration in the opposite side of the film is 4.53 × 10^−5^ g/cm^3^ (98% RH at 38 °C), then Δ*C* = 4.53 × 10^−5^ g/cm^3^. For *WVTR* = *J*/*A* and when combining Equations (2) and (3), we can calculate the diffusion coefficient *D* (cm^2^/s) for every film as follows:

(3) $$D_{WV}=WVTR*\frac{\Delta x}{\Delta C}$$

where *WVTR* [g/(cm^2^·s)] is the water–vapor transmission rate, Δ*x* (cm) is the film thickness, and Δ*C* (g/cm^3^) is the humidity concentration gradient in the two opposite sides of the film. According to the mathematical derivation of the *D_wv_* factor from Equation (2), this engineering factor is independent from the tested film thickness.

### 4.8. Oxygen Transmission Rate Measurements and Oxygen Permeability Calculation

Briefly, films of each sample with a 10 cm diameter were placed on an Oxygen Permeation Analyzer (8001, Systech Illinois Instruments Co., Johnsburg, IL, USA). According to the ASTM D 3985 method, tests were carried out at 23 °C and 0% RH, and the Oxygen Transmission Rate (OTR) results were expressed as cc O_2_/m^2^/day. From the measured OTR values, the oxygen permeability coefficient values (PeO_2_) were calculated according to the method described in detail in previous publications [32,42]. According to this literature, the theory such calculations are based on is gas permeability through polymers, and the relevant equation is as follows:

(4) $$\frac{J}{A}={Pe}_{gas}*\frac{\Delta C}{\Delta x}$$

where *J*/*A* [mol/(cm^2^·s)] is the specific amount of gas that passes through the membrane, *Pe_gas_* (cm^2^/s) is the permeability coefficient, Δ*C* (mol/cm^3^ STP) is the pressure gradient in the two opposite sides of the membrane, and Δ*x* (cm) is the membrane thickness.

Following the mathematical transformations described in the above-mentioned literature, we arrive to the following equation:

(5) $${Pe}_{O2}=OTR*\Delta x$$

According to the mathematical derivation of the P_O2_ factor from Equation (4), this engineering factor is independent from the tested film thickness.

### 4.9. Total Antioxidant Activity of ALG/G/xAC and ALG/G/xTO@AC Films

The total antioxidant activity of all ALG/G/xHNT and ALG/G/xTO@HNT films was estimated according to the diphenyl-1-picrylhydrazyl (DPPH) method. For the experiments, a 40 ppm ethanolic solution of a DPPH stock solution was prepared. Inside a dark glass bottle 10 mL of the DPPH stock solution, 300 mg of each film was placed and incubated until an equilibrium was observed after 24 h. The absorbance at a 517 nm wavelength of the DPPH solution was measured in the beginning (0 h) and in the end (24 h) of the incubation using a Jasco V-530 UV-vis spectrophotometer. For comparison, the absorbance of 10 mL of an ethanolic DPPH solution without the addition of any film was measured at 517 nm and considered the blank sample.

The % antioxidant activity after the 24 h incubation of the films was calculated according to the following equation:

(6) $$\%\mathrm{Antioxidant}\;\mathrm{activity}=\frac{{\mathrm{Abs}}_{\mathrm{blank}}-{\mathrm{Abs}}_{\mathrm{sample}}}{{\mathrm{Abs}}_{\mathrm{blank}}}\cdot 100$$

### 4.10. Application of ALG/G/xHNT and ALG/G/xTO@HNT Films as Active Coatings for “Tsalafouti” Type Spreadable Cheese Shelf—Life

**Figure 6:**
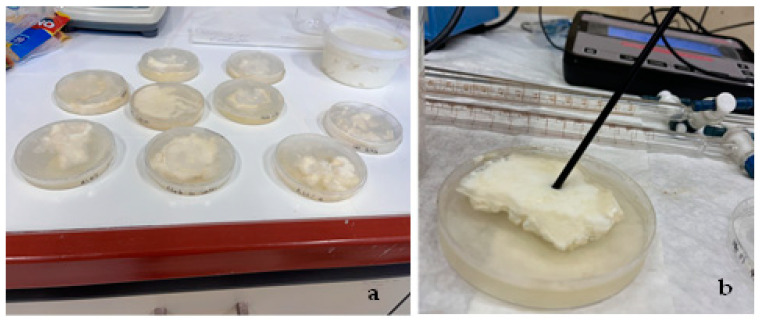
(**a**) “Tsalafouti” spread cheese uncoated and coated with two disc-shape films of all obtained ALG/G/xHNT and ALG/G/xTO@HNT samples as well as ALG/G films inside plastic Petri dishes, (**b**) the pH measurement of “tsalafouti”-coated samples during the preservation period of 12 days.

The commercial “Tsalafouti” cheese (produced by the “Papathanasiou” local dairy products factory) was purchased from a local market. When the packaging was opened, the “Tsalafouti” cheese was immediately used to make the coatings by adding 30 g of soft cheese inside plastic Petri dishes covered in their upper and bottom sides with two dried films of ALG/G/xHNT and ALG/G/xTO@HNT hydrogels each (see Figure 6a). The mesophilic microbial population in coated “Tsalafouti” cheese was assessed using PCA (Plate Count Agar). Microbiological analysis was performed for the total count of mesophilic bacteria. In accordance with the findings of Pala et al. [44] and Evert et al. [45] on Ricotta fresh cheese, the microbial analysis on Mesophilic bacteria in “Tsalafouti” soft cheese validates that such a microbial metric serves as a robust indicator of such traditional cheese shelf life. Briefly, the coating materials were first left to dry in sterile Petri dishes (both in the dish and the lid) at 30 °C for 48 h. Subsequently, 30 g of the “Tsalafouti” cheese was measured and positioned within the Petri dishes, creating a sandwich-like arrangement, aiming to achieve a uniform coating; this was done under sterile conditions (see Figure 6a). The coated cheese samples were then stored at a consistent temperature of 8 °C until the time of assessment. Intermediate samples of “Tsalafouti” cheese were taken randomly every 3 days for microbiological analysis and for a period of 12 days. For each measurement day, the corresponding Petri dish was removed, and 15 g of the cheese was transferred into 90 mL of sterile peptone water. The mixture underwent homogenization for a precise 5 min duration to obtain a homogeneous blend. Post homogenization, the blend was transferred to 9 mL tubes for dilution. Subsequently, 0.1 mL of the diluted cheese blend was drawn using a sterile pipette and evenly spread on a PCA agar plate with a sterile cell spreader. These Petri dishes were incubated at 35 °C for a 48 h duration. After the incubation period, microbial colonies were enumerated, and the colony counts were converted to log10 for analysis. Through this process, the microbial population in the coated “Tsalafouti” cheese samples was thoroughly evaluated, providing insights into the efficacy of the coatings for microbial control. For comparison, the pH of all coated and uncoated samples was monitored every 3 days during the preservation period of 12 days (see Figure 6b).

### 4.11. Statistical Analysis

The statistical analysis of the data of the microbial tests corroborates the findings as being statistically significant at a 5% level (*p* ≤ 0.05), with a test statistic F equaling 2.683385 and a *p*-value of 0.0260449. Thus, the null hypothesis was rejected. Furthermore, the large effect size observed (0.89) indicates substantial and meaningful differences not attributable to random chance, while the η2 of 0.44 signifies that the group accounts for 44.3% of the variance from the average. This empirical evidence underscores the potential utility of various coatings for the effective control of bacterial proliferation, thereby enhancing the microbial quality of Tsalafouti cheese and potentially other cheese varieties.

Moreover, statistical analysis was performed on mechanical properties, the water/oxygen barrier, and antioxidant activity data, using the one-way analysis of variance (ANOVA) through SPSS software. The Tukey HSD test was employed to evaluate the significance of mean values. Significance was set at a 5% level (*p* ≤ 0.05) to determine statistically relevant differences. All data are presented as mean value ± standard deviation (SD). The measurements were conducted in at least three replications for consistency and reliability.

## Figures and Tables

**Table 1:** The calculated values of Young’s (E) Modulus, ultimate tensile strength (σ_uts_), and %ε strain at break.

Sample Code Name	E-Elastic Modulus (MPa)	σ_uts_ (MPa)	%ε
ALG/G	445.5 (63.8)	15.2 (2.4)	40.2 (4.7)
ALG/G/5HNT	25.4 (6.6)	2.4 (0.7)	86.9 (11.4)
ALG/G/10HNT	617.7 (20.3)	21.8 (1.9)	32.6 (5.8)
ALG/G/15HNT	621.3 (59.5)	16.9 (3.5)	18.4 (6.7)
ALG/G/5TO@HNT	480.9 (20.1)	16.5 (0.9)	46.5 (6.4)
ALG/G/10TO@HNT	607.3 (53.4)	19.2 (1.6)	36.8 (4.1)
ALG/G/15TO@HNT	628.9 (66.6)	18.5 (1.1)	33.4 (3.9)

**Table 2:** The film thickness, water vapor transmission rate (*WVTR*), water diffusivity (D), oxygen transmission rate (OTR), and oxygen diffusivity (Pe_O2_) values of pure ALG/G film as well as all ALG/G/xHNT and ALG/G/xTO@HNT films.

Sample Code Name	Film Thickness (mm)	Water Vapor Transmission Rate·10^−6^ (*WVTR*) (g∙cm^−2^∙day^−1^)	Water Vapor Diffusion Coefficient *D_wv_* (10^−4^ cm^2^∙s^−1^)	Film Thickness (mm)	Oxygen Transmission Rate (OTR) (mL∙m^−2^∙day^−1^)	Oxygen Permeability Pe_O2_ 10^−6^ (cm^2^∙s^−1^)
ALG/G	0.15 ± 0.01 ^a,c^	2.46 ± 0.12 ^a^	8.69 ± 0.11 ^a^	0.15 ± 0.01 ^a^	196459 ± 185 ^a^	3.41 ± 0.31 ^a^
ALG/G/5HNT	0.16 ± 0.01 ^a,b^	2.57 ± 0.10 ^b^	9.69 ± 0.09 ^b^	0.22 ± 0.01 ^b^	134254 ± 124 ^b^	3.38 ± 0.32 ^a^
ALG/G/10HNT	0.17 ± 0.01 ^b^	2.09 ± 0.13 ^c^	8.39 ± 0.12 ^c^	0.17 ± 0.01 ^c^	82374 ± 84 ^c^	1.60 ± 0.16 ^b^
ALG/G/15HNT	0.16 ± 0.01 ^a,b^	2.14 ± 0.09 ^d^	8.07 ± 0.08 ^d^	0.13 ± 0.01 ^d^	192154 ± 186 ^a^	2.95 ± 0.28 ^c^
ALG/G/5TO@HNT	0.15 ± 0.01 ^a,c^	2.17 ± 0.07 ^e^	7.67 ± 0.06 ^e^	0.15 ± 0.01 ^a^	125335 ± 112 ^d^	2.14 ± 0.19 ^d^
ALG/G/10TO@HNT	0.14 ± 0.01 ^c^	2.16 ± 0.07 ^e^	7.13 ± 0.05 ^f^	0.33 ± 0.01 ^e^	34984 ± 54 ^e^	1.33 ± 0.21 ^e^
ALG/G/15TO@HNT	0.14 ± 0.01 ^c^	2.16 ± 0.05 ^e^	7.11 ± 0.04 ^f^	0.20 ± 0.01 ^f^	124256 ± 123 ^d^	2.91 ± 0.29 ^c^

Results are expressed as mean ± standard deviation (n = 12 for thickness, n = 3 for *WVTR* and OTR). Means in the same column bearing same superscript letters, i.e., a, b, c, d, e, f, are significantly equal (*p* < 0.5).

**Table 3:** The pH Values of Tsalafouti Soft Cheese During Storage at 8 °C for 12 Days after the application of different coatings.

Coating Type	Day 0	Day 3	Day 6	Day 9	Day 12
Uncoated	4.59 ± 0.10 ^a^	4.544 ± 0.12 ^b^	4.453 ± 0.11 ^c^	4.409 ± 0.13 ^f^	4.315 ± 0.15 ^h^
ALG/G	4.59 ± 0.05 ^a^	4.550 ± 0.09 ^b^	4.504 ± 0.07 ^d^	4.465 ± 0.08 ^c,j^	4.383 ± 0.10 ^i^
ALG/G/5HNT	4.59 ± 0.12 ^a^	4.578 ± 0.14 ^a^	4.542 ± 0.13 ^b^	4.492 ± 0.12 ^c^	4.443 ± 0.11 ^j^
ALG/G/10HNT	4.59 ± 0.07 ^a^	4.579 ± 0.05 ^a^	4.541 ± 0.09 ^b^	4.531 ± 0.10 ^e^	4.448 ± 0.08 ^j^
ALG/G/15HNT	4.59 ± 0.15 ^a^	4.581 ± 0.11 ^a^	4.553 ± 0.10 ^b^	4.542 ± 0.12 ^b^	4.461 ± 0.14 ^j^
ALG/G/5TO@HNT	4.59 ± 0.11 ^a^	4.579 ± 0.13 ^a^	4.533 ± 0.08 ^e^	4.521 ± 0.09 ^g^	4.453 ± 0.07 ^j^
ALG/G/10TO@HNT	4.59 ± 0.13 ^a^	4.583 ± 0.07 ^a^	4.571 ± 0.15 ^a^	4.531 ± 0.11 ^e^	4.457 ± 0.09 ^j^
ALG/G/15TO@HNT	4.59 ± 0.09 ^a^	4.584 ± 0.08 ^a^	4.575 ± 0.05 ^a^	4.532 ± 0.14 ^e^	4.471 ± 0.10 ^k^

Results are expressed as mean ± standard deviation (n = 3). Means bearing the same superscript letters, i.e., a, b, c, d, e, f, g, h, i, j, k are significantly equal (*p* < 0.5).

## Data Availability

The datasets generated for this study are available on request to the corresponding author.
